# Return to Work Guidelines Following Neurosurgical Procedures

**DOI:** 10.7759/cureus.11982

**Published:** 2020-12-08

**Authors:** Gina N Guglielmi, Jason M Seibly

**Affiliations:** 1 Neurosurgery, Carle BroMenn Medical Center, Bloomington, USA; 2 Neurosurgery, Central Illinois Neuroscience Foundation, Bloomington, USA

**Keywords:** return to work, return to activity

## Abstract

Recovery time following surgical procedures is a consideration every practicing surgeon must deliberate upon throughout his or her career. The decision to restrict patients from returning to work or various activities encountered on a daily basis following an operation is dependent on many factors. Surgeons must take into account patient population, individual comorbid conditions, complexity and length of surgery, immediate postoperative course, and baseline functional abilities. Thus, returning to work and various activities, including physical activity, work-related activity, and recreational activity alike, following invasive procedures is individualized from patient to patient. Most spinal procedures are performed by neurosurgeons or orthopedic surgeons. This article suggests a framework to guide appropriate return to work and activity escalation time frames following various spinal procedures.

## Introduction

The focus of this paper revolves around the opinions of orthopedic surgeons and neurosurgeons. However, any surgeon operating on the spine will need to determine when postoperative patients may return to normal activities. The rehabilitation process in the postsurgical patient involves slow progression of activity levels until normalcy is achieved. The surgeon must weigh the risks of injuring the operative site by allowing too rapid progression of activity following surgery to the benefits of the patient's return to enjoyable recreational activities. Maximizing patient recovery in a timely manner aided by appropriate activity is essential during the rehabilitation period. In many cases, patients are reliant on their income and are eager to return to work in a quicker time frame than may be deemed reasonable by the surgeon. If a patient’s activity is increased too abruptly, there is potential for increased pain, muscle spasm/injury, wound dehiscence, recurrent disc herniation, or hardware failure, among other complications. However, none of these complications has been quantified. Most postoperative physical restrictions are anecdotal and based on theoretical risks. The majority of studies on postoperative complications, such as recurrent disc herniations, consider patient characteristics such as age, gender, body mass index (BMI), diabetes, smoking status, or radiographic findings [1,2]. Few studies, if any, analyze postoperative complications in relation to activity levels following surgery. Surgeon recommendations are diverse, and, to date, there have been no published guidelines in the spinal or neurosurgical literature as to when it is acceptable to return uncomplicated, postoperative patients to various levels of activity.

## Materials and methods

**Figure 1:**
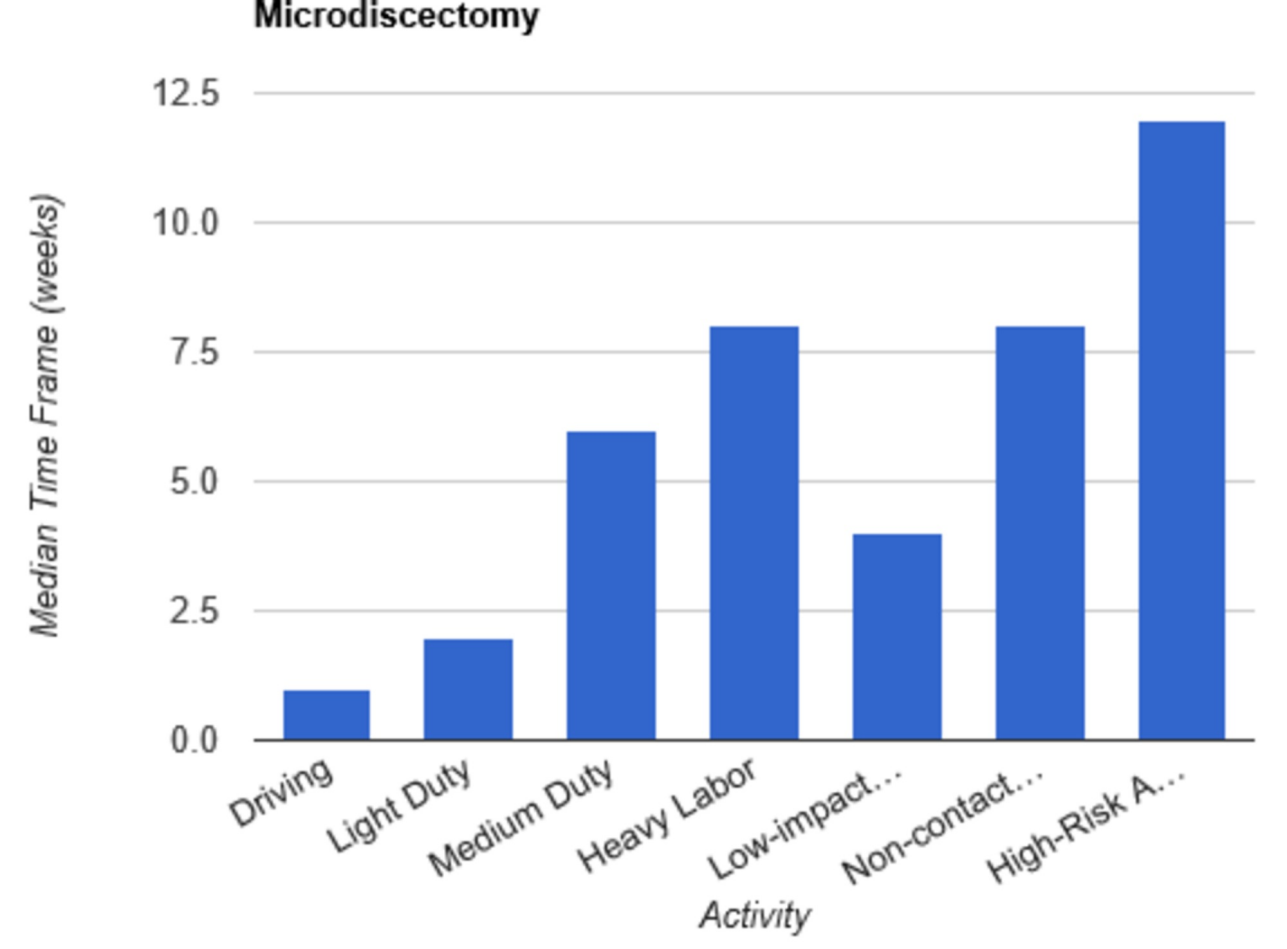
Return to Activity Following Microdiscectomy low-impact…, low-impact exercise; non-contact…, non-contact sports; high-risk…, high-risk activities or contact sports

**Figure 2:**
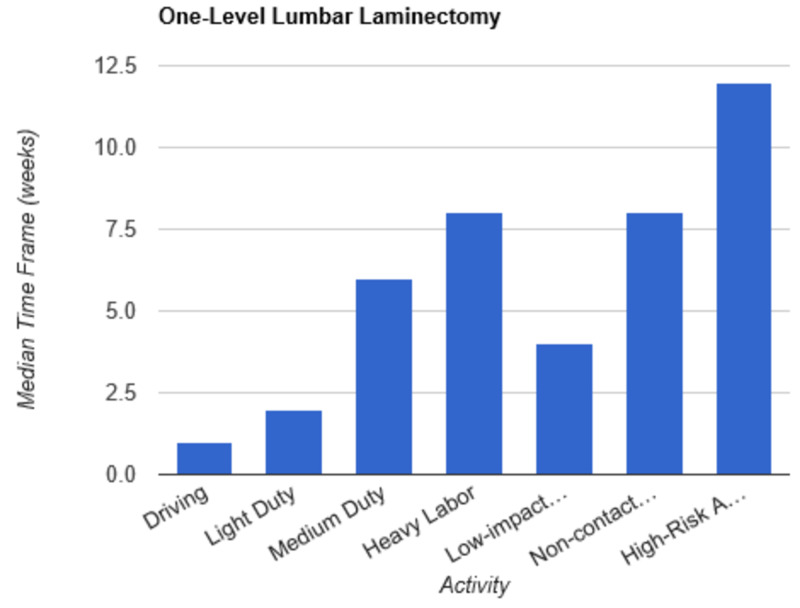
Return to Activity Following One-Level Laminectomy low-impact…, low-impact exercise; non-contact…, non-contact sports; high-risk…, high-risk activities or contact sports

**Figure 3:**
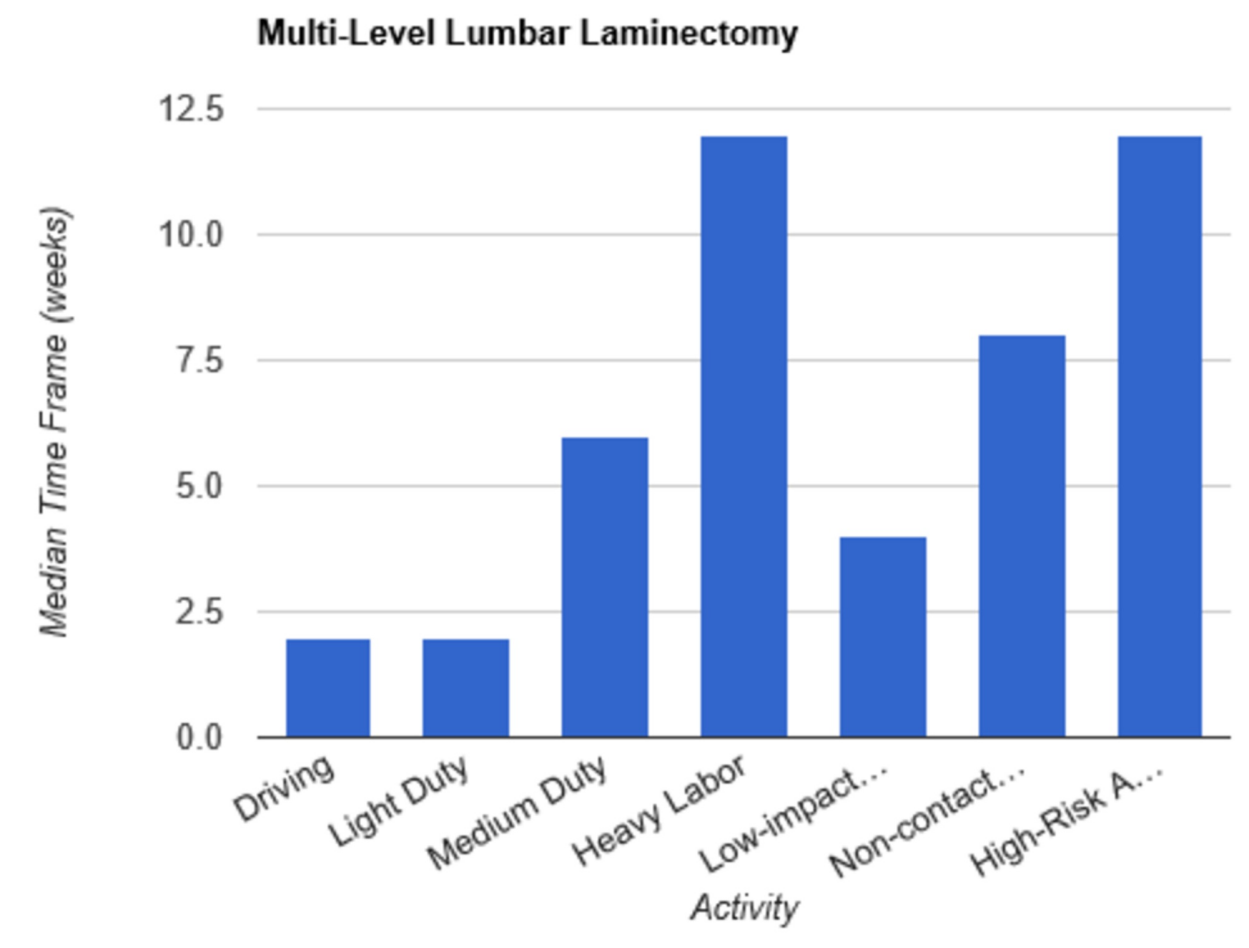
Return to Activity Following Multi-Level Laminectomy low-impact…, low-impact exercise; non-contact…, non-contact sports; high-risk…, high-risk activities or contact sports

**Figure 4:**
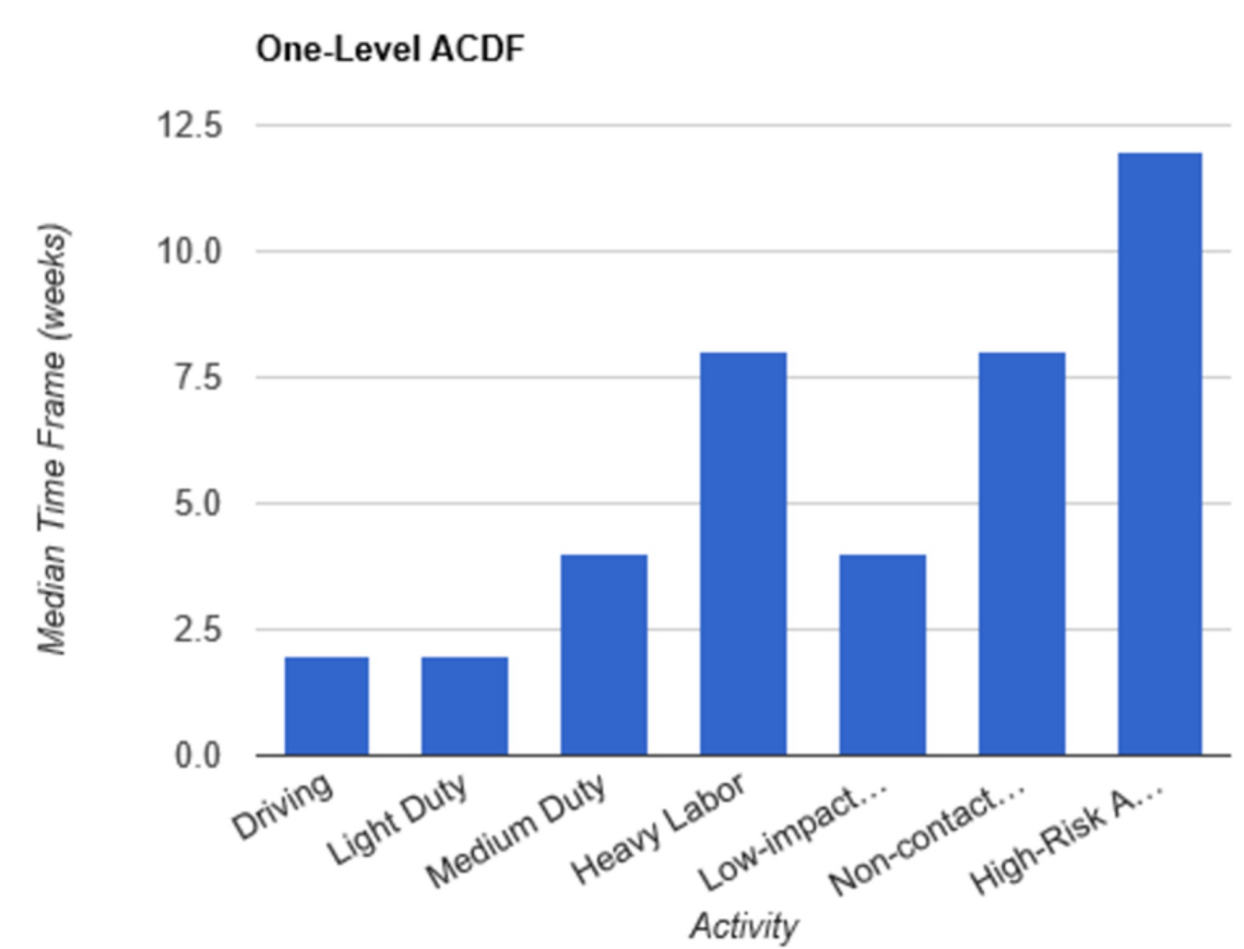
Return to Activity Following One-Level ACDF low-impact…, low-impact exercise; non-contact…, non-contact sports; high-risk…, high-risk activities or contact sports; ACDF, anterior cervical discectomy and fusion

**Figure 5:**
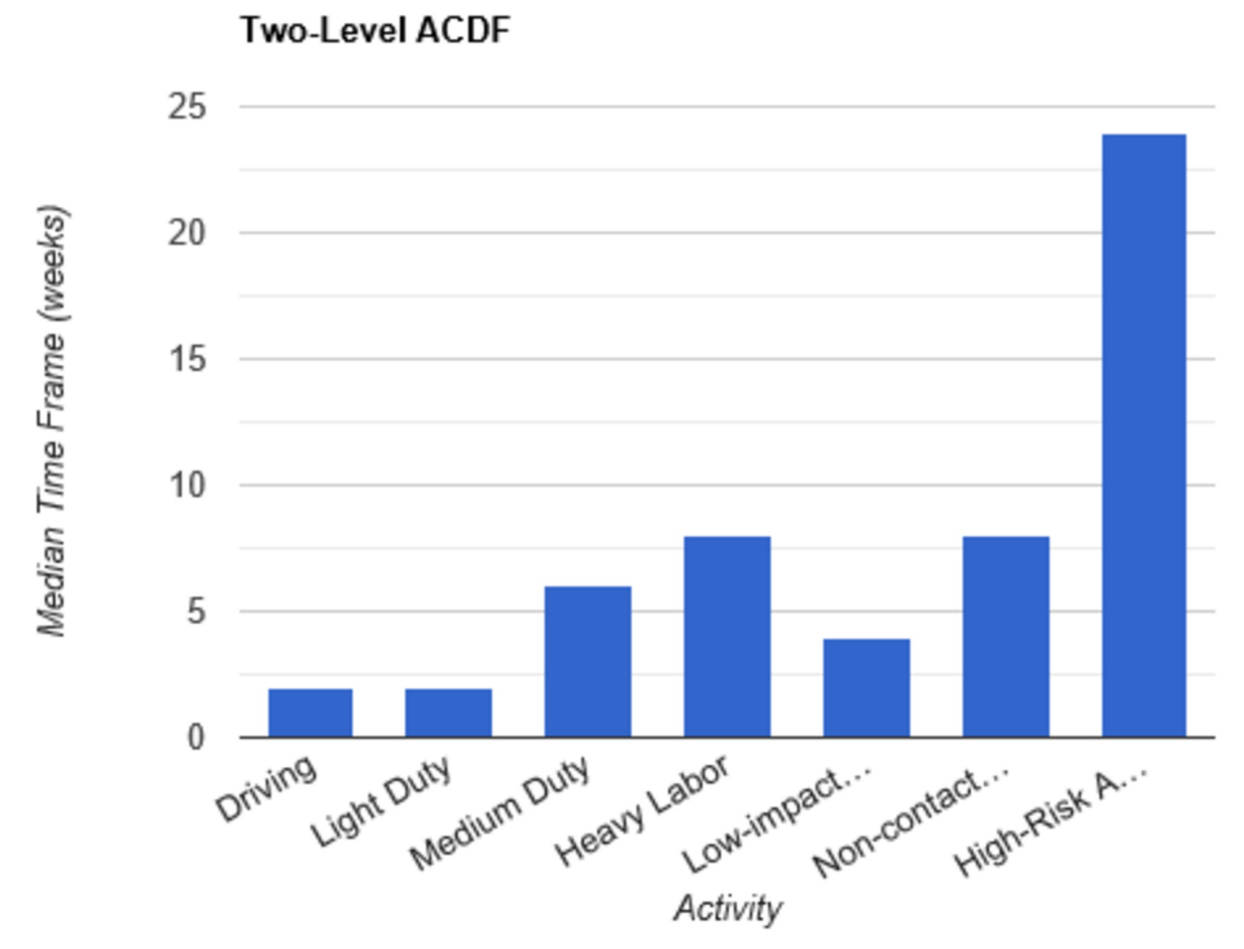
Return to Activity Following Two-Level ACDF low-impact…, low-impact exercise; non-contact…, non-contact sports; high-risk…, high-risk activities or contact sports; ACDF, anterior cervical discectomy and fusion

**Figure 6:**
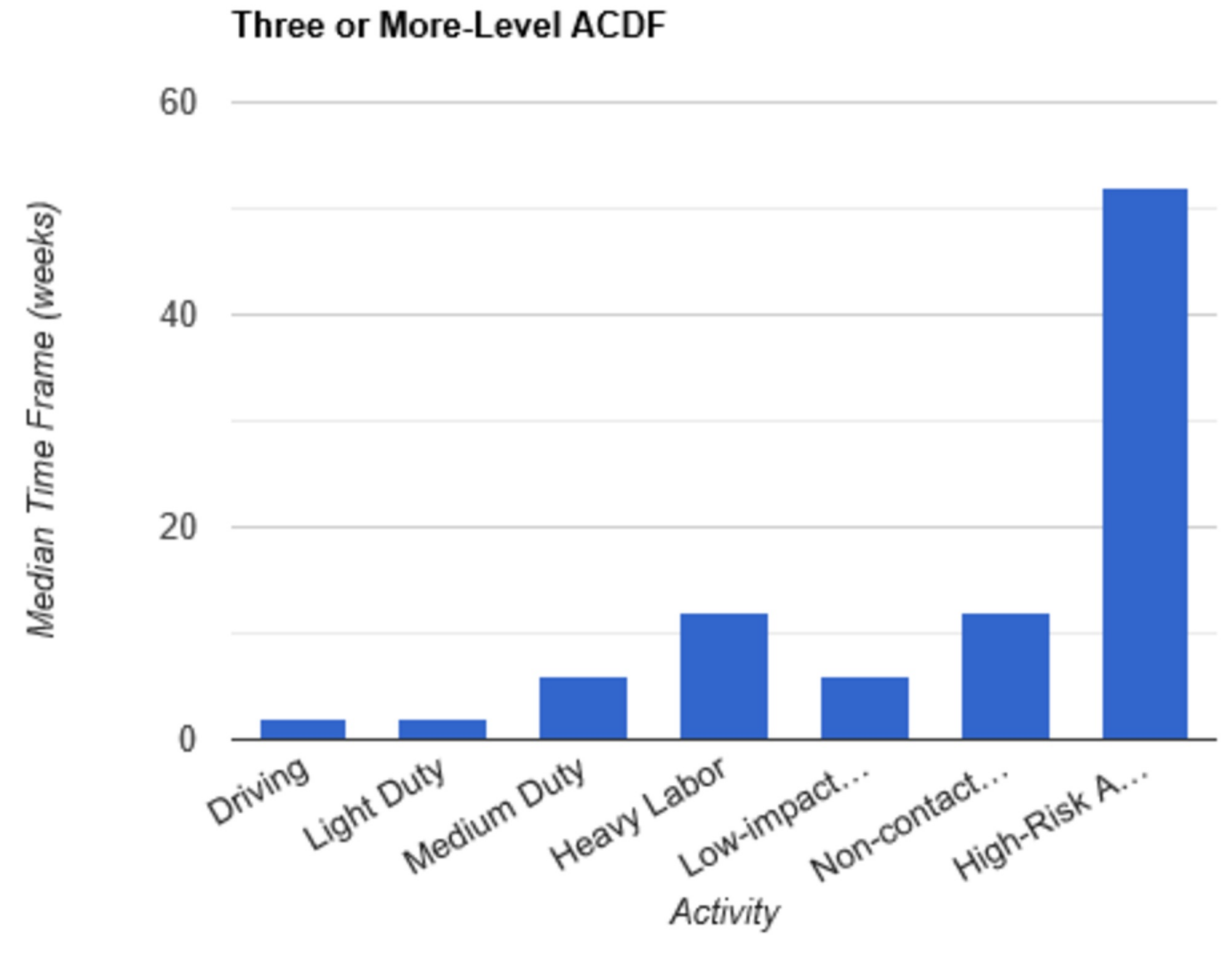
Return to Activity Following Three or More-Level ACDF low-impact…, low-impact exercise; non-contact…, non-contact sports; high-risk…, high-risk activities or contact sports; ACDF, anterior cervical discectomy and fusion

**Figure 7:**
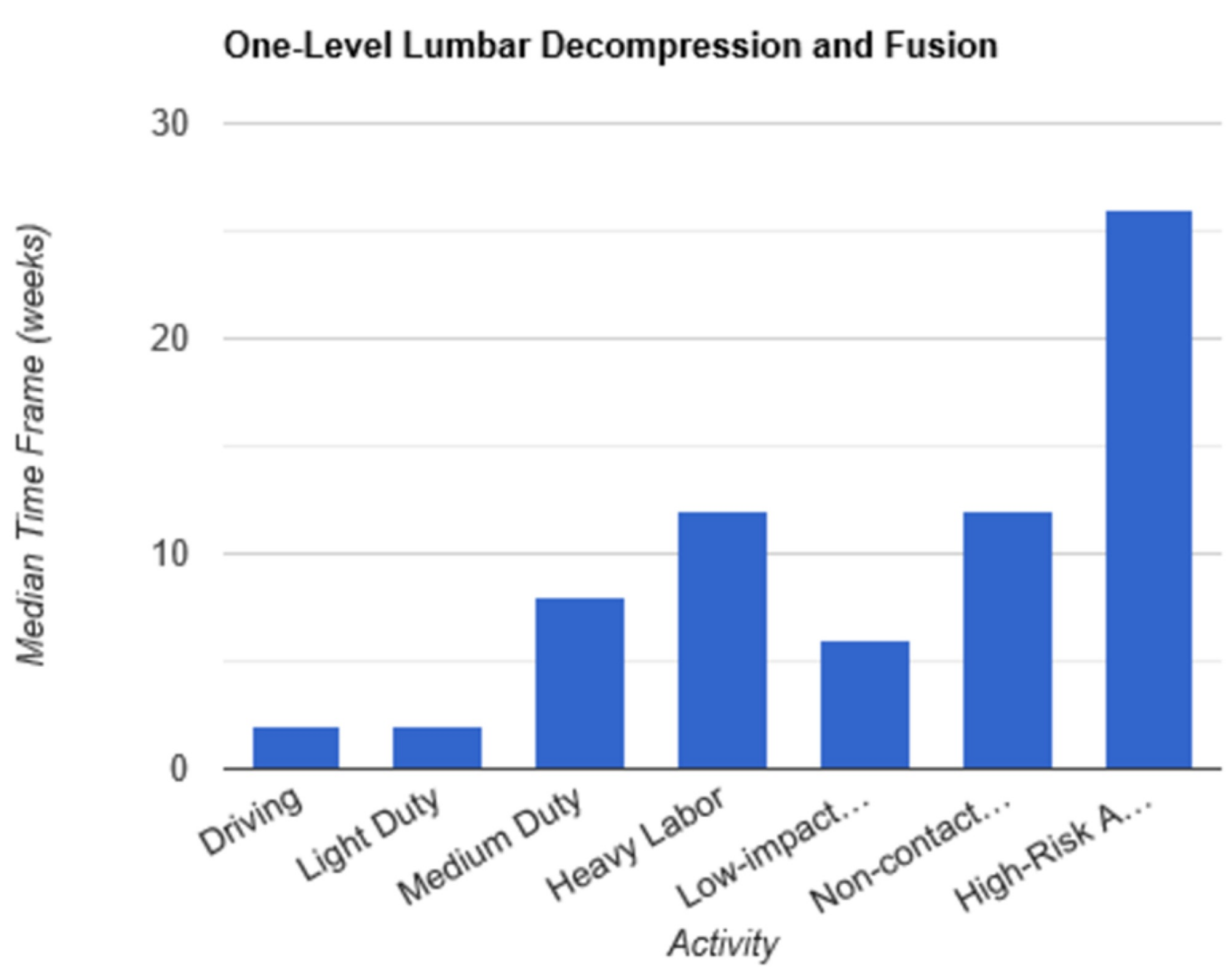
Return to Activity Following One-Level Lumbar Decompression and Fusion low-impact…, low-impact exercise; non-contact…, non-contact sports; high-risk…, high-risk activities or contact sports

**Figure 8:**
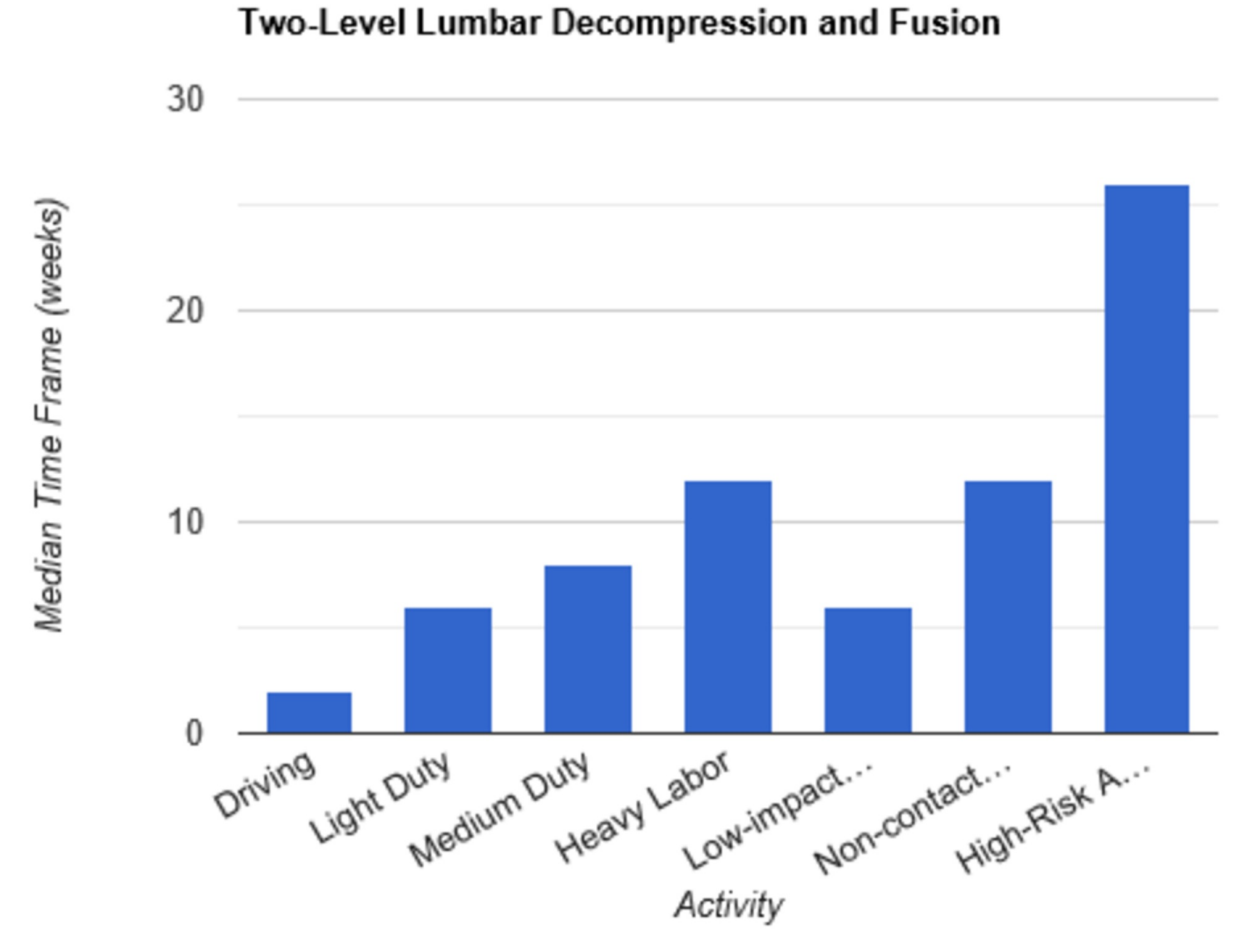
Return to Activity Following Two-Level Lumbar Decompression and Fusion low-impact…, low-impact exercise; non-contact…, non-contact sports; high-risk…, high-risk activities or contact sports

**Figure 9:**
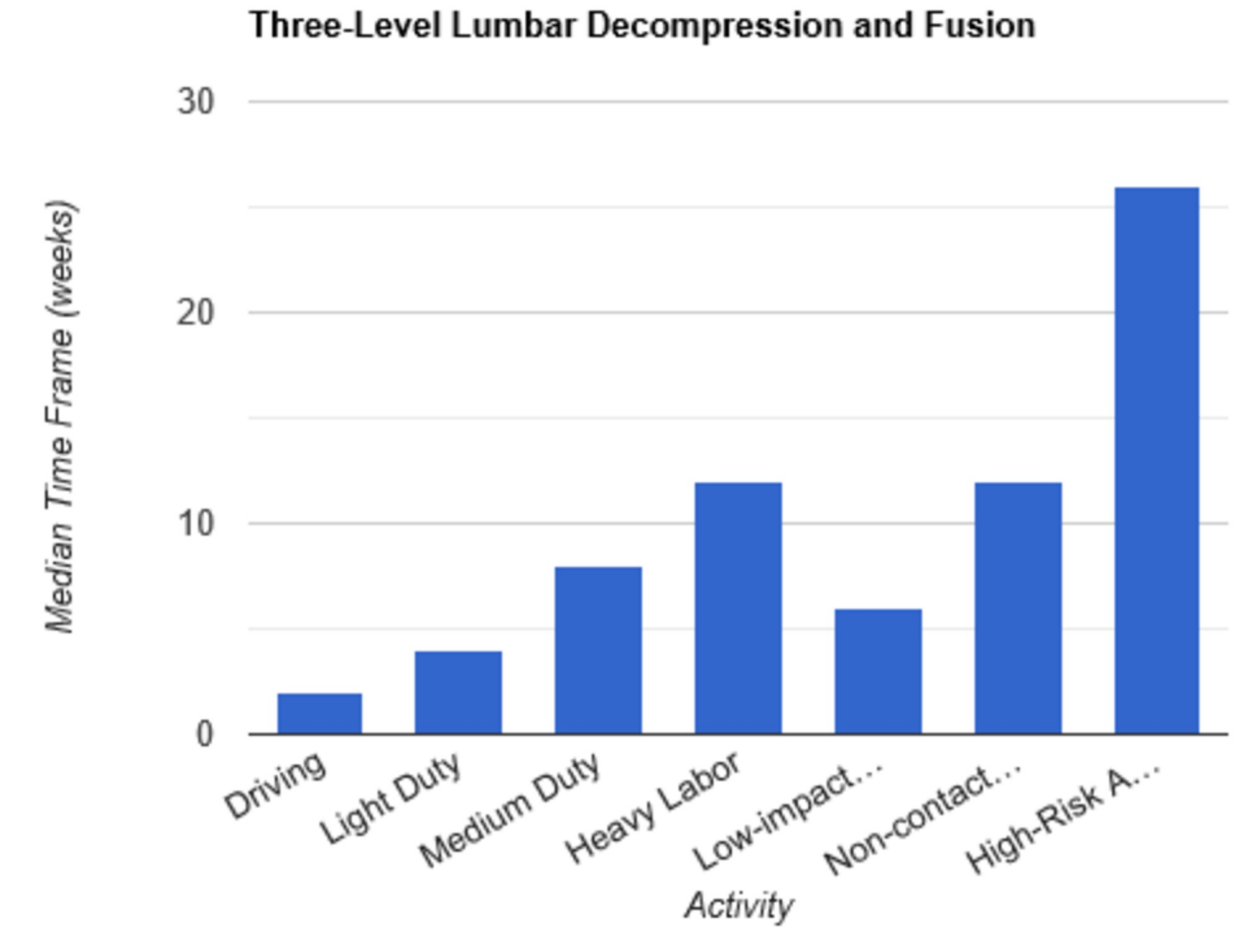
Return to Activity Following Three-Level Lumbar Decompression low-impact…, low-impact exercise; non-contact…, non-contact sports; high-risk…, high-risk activities or contact sports

**Figure 10:**
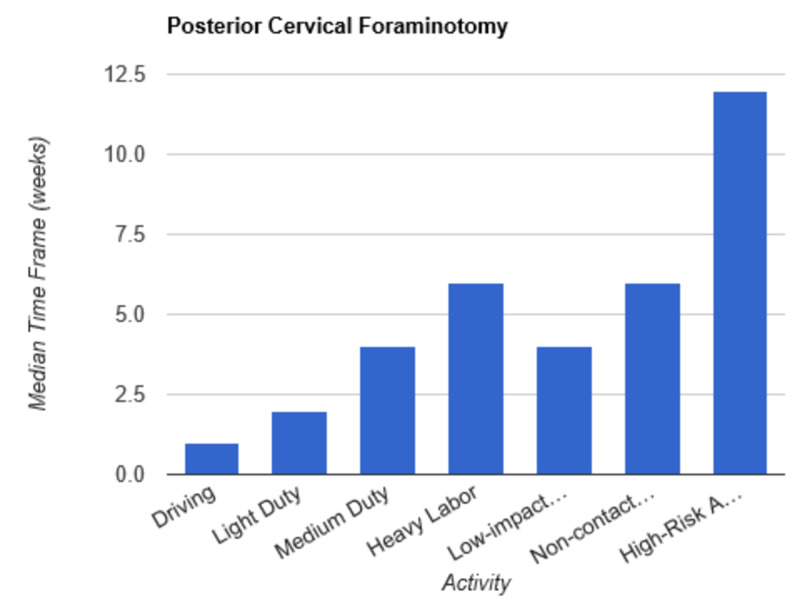
Return to Activity Following Posterior Cervical Foraminotomy low-impact…, low-impact exercise; non-contact…, non-contact sports; high-risk…, high-risk activities or contact sports

In order to determine consistent postoperative recommendations, a group of 56 surgeons who routinely perform spinal surgeries were asked to provide their personal time-off restrictions for various activity levels for several different spinal procedures. The results were collected over the course of one month. The goal of this survey is to determine an acceptable time frame for a postoperative patient to resume various activities of daily living. This questionnaire assumes that the surgery and perioperative course were routine and without complication. Each surgeon was asked a series of 10 questions, broken down by specific procedure types: microdiscectomy, one-level lumbar laminectomy, multi-level lumbar laminectomy, one-level anterior cervical discectomy and fusion (ACDF), two-level ACDF, three or more level ACDF, one-level lumbar instrumented fusion, two-level lumbar instrumented fusion, three or more level lumbar instrumented fusion, and posterior cervical foraminotomy. Various levels of activity were provided, which included driving off pain medications, light duty/clerical work, medium duty including occupations such as that of a nurse and truck/fork-lift driver, heavy labor including occupations such as construction and bricklaying, low-impact exercises including stationary bikes or elliptical, non-contact sports including softball, tennis, and weight-lifting, and high-risk activities or contact sports including roller coasters and playing football. Based on individual preference, the surgeon selected a specified time frame postoperatively in which they would allow their patients to resume each of the aforementioned activities. The participating surgeon was also given the option to never allow return of their patient to the activity presented. Figures 1-10 outline the list of activity levels and the median time frames selected by surgeons. In Table 1, both both time frames were provided in areas where there was equal division of time frames selected by surgeons for any given activity.

 

**Table 1:** Return to Work and Activity: Median Time Frames Following Neurosurgical Procedures ACDF, anterior cervical discectomy and fusion; TLIF, transforaminal lumbar interbody fusion

	Microdiscectomy	One-level lumbar laminectomy	Multi-level lumbar laminectomy	One-level ACDF	Two-level ACDF	Three or more level ACDF	One-level lumbar fusion/TLIF	Two-level lumbar fusion/TLIF	Three or more level lumbar fusion/TLIF	Posterior cervical foraminotomy	
Driving off pain medications	1 week	1 week	2 weeks	2 weeks	2 weeks	2 weeks	2 weeks	2 weeks	2 weeks	1 week	
Light duty, clerical work	2 weeks	2 weeks	2 weeks	2 weeks	2 weeks	2 weeks	2 weeks	6 weeks	4 weeks	2 weeks	
Medium duty: nurse, truck, fork-lift driver	6 weeks	6 weeks	6 weeks	4-6 weeks	6 weeks	6 weeks	8 weeks	8 weeks	8 weeks	4 weeks	
Heavy labor: construction, bricklaying	8 weeks	8 weeks	3 months	8 weeks	8-12 weeks	3 months	3 months	3 months	3 months	6 weeks	
Low-impact exercise: stationary bike or elliptical	4 weeks	4 weeks	4 weeks	4 weeks	4 weeks	6 weeks	6 weeks	6-8 weeks	6-8 weeks	4 weeks	
Non-contact sports: softball, tennis, weight-lifting	8 weeks	8 weeks	8 weeks	8 weeks	8 weeks	3 months	3 months	3 months	3 months	6 weeks	
High-risk activities or contact sports: roller coaster, football	3 months	3 months	3 months	3 months	6 months	12 months	6 months	6-12 months	6-12 months	3 months	

## Results

The 56 spine surgeons provided feedback regarding individual preferences on return to routine and recreational activities following spine surgery. Surgeons indicated allowable return to work and activity time frames while taking into account the surgery performed. All participants were board-certified, board-eligible neurosurgeons or fellowship-trained spine surgeons. At the time the survey was conducted, all participants were actively working in the United States of America. Data were collected and results were analyzed based on individual surgery. The data are presented based on the average time frame acceptable by participating surgeons for returning to each activity in the postoperative course.

Following a routine microdiscectomy, one-level laminectomy, and posterior cervical foraminotomy, driving could be safely resumed on average one week postoperatively. Surgeons recommended waiting two weeks to return to driving following the remainder of procedures included in the survey. Light duty, including clerical work, was acceptable two weeks following any procedure, with the exception of waiting four weeks following a three or more level fusion and six weeks following a two-level fusion.

Medium duty, including occupations such as nursing, truck drivers, and fork-lift drivers, demonstrated more variability in responses. Six weeks of recovery was recommended before returning to work following a microdiscectomy, one- or multi-level laminectomy, and two- or three-level ACDF. Following a posterior cervical foraminotomy, surgeons felt patients could safely return to medium duty after four weeks. After a one-level ACDF, four to six weeks was recommended. The greatest amount of time was recommended following all lumbar fusions, with surgeons recommending on average a total of eight weeks of recovery prior to returning to medium duty.

Heavy labor was defined as occupations involving construction or bricklaying. Following a posterior cervical foraminotomy, an average of six weeks’ recovery was recommended prior to return to work. Eight weeks was recommended following a microdiscectomy, one-level laminectomy, or one-level ACDF. A range of 8 to 12 weeks was recommended following a two-level ACDF. The remainder of procedures, including multi-level laminectomy, three or more level ACDF, and all lumbar fusions, shared the recommendation of waiting three months postoperatively prior to returning to work.

Low-impact exercises, such as a stationary bike or elliptical, could be resumed on average of four weeks following a microdiscectomy, one- or multi-level laminectomy, one- or two-level ACDF, or posterior cervical foraminotomy. Six weeks was recommended following a three or more level ACDF and one-level lumbar fusion. Surgeons recommended waiting six to eight weeks following lumbar fusions involving two or more levels prior to returning to low-impact exercises.

Non-contact sports, including tennis, softball, and weight-lifting, could be safely resumed after six weeks following a posterior cervical foraminotomy. Surgeons recommended waiting an average of eight weeks following a microdiscectomy, one or multi-level laminectomy, and one or two-level ACDF. The average time frame following a three or more-level ACDF and any lumbar fusion was three months on average.

High-risk activities or contact sports such as roller-coasters and football, respectively, were recommended on average to resume after three months following a microdiscectomy, one- or multi-level laminectomy, one-level ACDF, or posterior cervical foraminotomy. Six months was recommended to recover prior to returning to high-risk activities or contact sports following a two-level ACDF and one-level lumbar fusion. The average time frame was extended for both two- and three-level lumbar fusions to 6 to 12 months. Interestingly, surgeons recommended waiting one year prior to returning to high-risk activities following a three or more level ACDF.

## Discussion

Our results indicate consensus among physicians that driving and light-duty can be safely recommended in the first one to two weeks following a routine microdiscectomy, one-level lumbar laminectomy, multi-level lumbar laminectomy, one-level ACDF, two-level ACDF, three or more level ACDF, one, two, and three or more level lumbar instrumented fusions, and posterior cervical foraminotomy. As the complexity of the surgery increased, surgeons appeared to favor extended periods of time prior to allowing resumption of both heavy labor and non-contact sports. Return to medium duty fell somewhere in the interim. There were two instances when a single surgeon recommended against allowing a patient to return to high impact activities at any time point: following a two-level ACDF and following a one-level lumbar instrumented fusion. Five participants recommended never returning to high-risk activities following a two-level lumbar instrumented fusion. Seven participants recommended never returning to high-risk activities following a three or more level lumbar instrumented fusion. Discrepancy among surgeons was demonstrated with regard to return to low-impact exercise following a two-level ACDF. An equal number of participants recommended return after two weeks and three months. This trend was also demonstrated with regard to returning to medium duty following a one-level lumbar instrumented fusion. In this instance, an equal number of participants recommended return to medium duty at both six weeks and three months. Additionally, an equal number of surgeons recommended return to non-contact sports at six weeks and three months following posterior cervical foraminotomy.

Based on the collected information, the authors would recommend establishing these time frames for advancing the postoperative patient’s activity. Obviously, all patients should be treated individually, and this recommendation may not fit everyone. It can, however, serve as a starting point for patients with acceptable surgical risk who had an uncomplicated intraoperative and immediate postoperative course. There is also a difference between physical ability to return to work versus “safe” behaviors based on the surgery performed. A common example is following a microdiscectomy. There are many instances when a patient awakes from surgery with complete resolution of preoperative symptoms. Simple activities such as sitting, walking, or sleeping, which may have proved troublesome prior to the operation, are performed with ease. Although the patient may feel physically able to return to work or recreational activities, there is still the risk of recurrent disc herniation. Therefore, patients are routinely advised to avoid excessive bending, twisting, and lifting activities immediately postoperatively.

Another consideration is the patient’s physical and/or emotional ability to reach their postoperative goals in returning to the workplace. It is well established that there are patient and occupational factors that affect their ability to return to work. One study analyzed occupational characteristics that affected an employed patient's ability to return to work after lumbar spine surgery. They concluded that despite a favorable surgical outcome, 85.3% of patients were able to return to work at one year and 7% failed to ever return to work. Some of the factors that negatively impacted the patient's ability to return to work were female gender, higher back pain score as measured using the Oswestry Disability Index (ODI), longer duration of symptoms, more physically demanding occupations, worker’s compensation, and those on short-term disability at the time of surgery [3]. These predictors of a lower likelihood of return to work are consistently identified throughout several other investigations [4,5]. Specific to cervical spine surgery, three to four levels of fusion and the presence of myelopathy also diminish the possibility of a patient returning to the desired activity level [4]. In 2015, Nair et al. reported similar results after looking at elite athletes. In their review paper, 75% to 100% of patients were able to return to competitive play after a lumbar microdiscectomy. The time to return to play ranged from 2.8 to 8.7 months [5].

Return to play for different sports after total lumbar disc replacement has also been questioned in one study, with sporting activity being resumed between three and six months postoperatively [6]. Approximately 94.9% of patients were able to resume sports. Athletic performance improved in 84.6% of patients, and peak performance occurred approximately 5.2 months after surgery. They documented implant subsidence in 30% of patients, but this did not appear to require reoperation or affect outcome [6]. Due to the potential risk of spinal cord injury, return to play after cervical spine surgery tends to be more cautious. This is especially the case regarding contact sports such as football. Maroon et al. recommended in 2013 that professional athletes could resume contact sports after a single-level ACDF if the patient has a normal neurological examination, demonstrated full range of neck motion, and demonstrated a solid radiographic fusion [7]. A prospective study from Germany evaluated return to sports after surgical treatment of a lumbar disc herniation [8]. They reported that 91.4% of patients were able to return to sports. The mean time until return to play was 5.8 months after surgery; the range was 6 weeks to 24 months. They also reported a recurrent herniation rate of 5.7%, half of which occurred before the patient returned to sporting activity [8]. Ultimately, each patient’s treatment should be individualized, and it is up to the physician to make the determination when each patient can resume normal activities based on their particular situation.

## Conclusions

Returning to work following invasive procedures is a common concern for patients who undergo both routine neurosurgical and orthopedic surgeries. Complications can arise at any time in the intraoperative and postoperative course, which can alter the time an individual may be required to refrain from returning to the workplace or daily activities. Nevertheless, every patient must be individualized and respective considerations must be taken into account when deciding appropriate time frames for return to work and various activities encountered on a daily basis. Neurosurgeons and orthopedic surgeons alike may utilize these expert opinions to assist in the return of their surgical patients to various levels of activity following uncomplicated spinal procedures.
